# Health Experiences Research as a Resource and Mechanism for Veteran Engagement in VA Healthcare Research and Care Delivery

**DOI:** 10.1007/s11606-021-07306-2

**Published:** 2022-03-29

**Authors:** Shannon M. Nugent, Erika Cottrell, Sara J. Knight, Mark Helfand

**Affiliations:** 1 VA Portland Health Care System, Center to Improve Veteran Involvement in Care, R&D66, 3710 SW US Veterans Hospital Road, Portland, OR 97239 USA; 2 Oregon Health and Science University, Portland, OR USA; 3 VA Salt Lake City Health Care System, Salt Lake City, UT USA; 4 Department of Internal Medicine, University of Utah, Salt Lake City, UT USA

**Keywords:** Veteran engagement, health experiences research, qualitative research

## Abstract

Engaging patients in the research process helps to ensure researchers ask meaningful questions and generate useful evidence to inform healthcare decisions. In 2015, the Veterans Health Administration (VA) Health Services Research & Development (HSR&D) service convened a Veteran engagement workgroup, comprised of researchers, clinicians, and Veterans, to identify ways to integrate Veteran engagement into HSR&D. A subgroup was designated to explore the utility of health experiences research (research focused on enhancing understanding of people’s experiences with healthcare and illnesses) as a mechanism to complement and broaden traditional engagement mechanisms. The subgroup recommended the VA adopt the Database of Individual Patient Experiences (DIPEx) methodology for conducting and disseminating health experiences research (HER). In this paper, we describe (1) the key components of the DIPEx approach, (2) how these components complement and broaden current methods of Veteran engagement, (3) an update on VA activities using the DIPEx approach, and (4) a roadmap for future VA HER activities.

## INTRODUCTION

Researchers and funding agencies increasingly recognize the critical importance of engaging patients in the research process.^1–8^ Engaging people with lived experience helps identify important questions and outcomes and ensure meaningful evidence generated is to inform healthcare decisions.^5, 8^ Active, sustained engagement improves dissemination of research and builds trust, especially among under-represented populations.^7, 8^ Patient engagement exists on a continuum from consultation to partnership, depending on the level of patient involvement.^2^ Research teams often employ multiple forms of engagement throughout a study.^5^ Common engagement approaches include establishing advisory and stakeholder panels to guide a research study, having patients as project consultants, and including patients as study team members.^2, 4, 9^

Engaging patients in research can be time-consuming and resource-intensive.^5^ Some have noted people’s ability to participate in engagement activities and share their views openly may be influenced by many factors including power differentials, education level, personality, and group dynamics.^1, 3^ Moreover, those who have the resources to engage in panels are typically more “well-connected, well-informed, and well-off,” which may lead to over- or under-representation of certain perspectives, or put patients in a position of representing the voices of an entire community.^6^ As a result, researchers have evaluated existing practices and explored innovative approaches for augmenting patient engagement.^5^

In 2015, the Veterans Health Administration (VA) Health Services Research & Development (HSR&D) convened a Veteran engagement workgroup, comprised of researchers, clinicians, and Veterans to identify ways to integrate Veteran engagement into HSR&D.^10^ A subgroup explored the utility of health experiences research (research focused on enhancing understanding of people’s experiences with healthcare and illnesses) as a way to complement traditional engagement methods “to reach parts of the experience that more structured methods cannot, among those who might otherwise not be heard.”^1^ This subgroup recommended the VA adopt a specific qualitative approach, the Database of Individual Patient Experiences (DIPEx), as the methodology for conducting and disseminating health experiences research (HER).^1^ DIPEx is a qualitative methodology seeking to *collect*, *preserve*, and *disseminate* rigorously conducted and analyzed interviews from a broad range of individuals to capture the “widest possible range” of healthcare experiences. The HER subgroup explored the value of developing a VA repository of DIPEx projects and a process for expanding the VA’s capacity to produce inclusive studies of Veterans’ experiences with a condition or a health service.^1, 10^ In this paper, we describe (1) the key components of the DIPEx approach, (2) how these components function as a form of Veteran engagement and as a unique source of data that can be used to complement and broaden methods of Veteran engagement, (3) an update on VA DIPEx activities, and (4) a roadmap for future VA DIPEx activities.

## OVERVIEW OF DIPEX AND ITS STRENGTHS AS AN ENGAGEMENT METHOD

DIPEx combines rigorous qualitative methods for exploring health experiences with a commitment to broad dissemination and future use. DIPEx was developed by a multidisciplinary team of investigators as an inclusive form of patient engagement. For each DIPEx project (“module”) on a health topic, researchers conduct in-depth interviews with a maximum variation sample of 35–50 people. Interviews begin by inviting the patient to tell the story of their health condition, without interruption, and are then followed with semi-structured questions for details. Interview transcripts are approved by the participant and preserved in a repository, available for future use upon request. While there have been other recently developed repositories,^11^ interviews conducted under DIPEx are rigorously analyzed, meaningfully organized, and broadly disseminated in peer-reviewed journals as well as on a public facing website (https://healthexperiencesusa.org/). For web-based dissemination, researchers create short topic summaries, derived from thematic analysis, describing people’s experiences with different aspects of their health condition (Table 1). Topic summaries are written in lay-language, are developed in collaboration with participants, and illustrated with participant-approved text, audio, and/or video clips from interviews. Each module convenes a panel of patient, clinical, and scientific advisors who meet regularly to provide feedback on all aspects of the project, including recruitment and sampling, interview guide development, coding and thematic analysis, and web-module production. Each step is an opportunity for the participant to engage in the research process, which enhances overall transparency.

**Table 1:** Example of Topics and Subtopics Included in the Gulf War Illness Module

Topics	Subtopics
Military context	Pre-deployment (e.g., training, lack of preparation, abrupt deployment) In-theater/combat experiences Transitioning home (health screen, challenges re-assimilating into civilian life, Rest of military career (non-Gulf War deployments)
When something first went wrong	Description of when Veteran first noticed something wrong with his/her health
Illness and symptom experiences	GWI or other medical or mental health conditions Symptom descriptions
Diagnostic Journey	Diagnostic process
Living with Gulf War Illness	Illness impact on life Comorbidities (physical and mental health)
Coping and support (non-medical)	Coping mechanisms Other Veteran services used Advocacy, seeking information, and engagement in research
Experiences seeking healthcare	Navigating healthcare systems (VA, private, military) VA care Barriers to care
Experiences with treatment	Types of treatments used (pharmacological and non-pharm, treatment adverse events) Description of treatment utility
Parallel life experiences	Relationship, hobbies, children/family, work/education
Insights and reflections	Lessons learned Needs and challenges of Gulf War Era Veterans Messages for others (VA, military, clinicians, family)
Recommendations for VA	Future research needs Clinical care and system level needs and recommendations

There are several key methodologic elements of the DIPEx approach that complement current VA engagement activities. First, DIPEx studies use maximum variation sampling to reach individuals representing different sociodemographic, geographic, and health condition–related factors. Intensive multimodal recruitment efforts are made through social advocacy, community engagement, word-of-mouth, and leadership and clinical partnerships with the goal of capturing a diverse range of health experiences, not just the most common or most compelling experiences. This approach lends itself to enhancing the voices of individuals who are under-represented in traditional research studies, and/or are less likely to be included by more traditional engagement mechanisms. The commitment to maximum variation enhances the generalizability and combats convenience sampling biases and tokenism that may be present with other forms of engagement such as advisory panels or consultation with a single Veteran.

Second, a key feature of the DIPEx approach is every participant begins their interview by recounting their narrative in its entirety without interruption, “focusing on the issues that are most important to them.”^12^ Inviting participants to share in-depth narratives with limited initial direction allows for a more complete understanding of the lived experience with less risk of researcher bias. This approach decreases issues with power dynamics or discomfort sharing in a group setting with multiple stakeholders. Within each narrative, individuals share nuanced descriptions of how their health condition intertwines with their families, clinicians, and healthcare systems. Collecting and preserving this information can provide insights into areas of future improvement such as caregiver support, patient-clinician communication, clinician education, and broader healthcare system design.

Third, and perhaps most importantly, a core feature of the DIPEx approach is the commitment to broad, multimodal dissemination. Researchers analyze and organize the 35–50 interviews on a health condition to produce a publicly available web-based module on https://healthexperiencesusa.org streamlining access to information. Participants, who are engaged throughout the module-development process, share messages, insights, and hopes during their interview, knowing their voices will be heard by others. In addition, de-identified transcripts of all modules conducted using DIPEx are preserved and entire collections of interviews on health topics are housed in a repository and available for secondary analysis, upon approval. The in-depth stories combined with the accessibility of a web-based module and the maintenance of the repository can offer the opportunity for less common insights and ideas to influence subsequent research questions, recruitment methods, intervention design, and modalities of disseminating findings.

There are several ways in which the DIPEx approach complements and broadens currently available VA tools for engagement and provides a resource. First, Veterans who are involved in the governance, leadership, or conduct of a research project as a team member, consultant, or advisor may be able to consult the organized and synthesized data in the DIPEx modules as a way to understand the broader experiences of Veterans, and serve as an interpreter, curator, and representative for a wider range of Veteran experiences. In addition, these Veterans may refer loved ones and community members to the DIPEx module on specific conditions, to engender social awareness, which may ultimately lead to social advocacy and policy change. The online and repository resources represent a lasting form of engagement that can be used as a tool for bringing the patient’s voice into clinical education and development of clinical strategies, guidelines, and quality improvement initiatives.^12–17^ Finally, researchers conducting studies in priority areas could use materials from DIPEx modules to understand Veteran perspectives, identify areas of need, and better articulate research questions.^18^ Others have suggested using data for DIPEx modules as preliminary data to justify and support future research and/or to inform the development of clinical trial recruitment strategies or the development of patient-reported outcome measures.^19^

Despite these advantages, the DIPEx approach is time-consuming and resource-intensive; it is not feasible for every research study to conduct a DIPEx module prior to beginning. However, once a DIPEx module on a particular topic is available, it can be used as a resource for multiple projects and initiatives. Ultimately, the HER subgroup recommended the VA set priorities for DIPEx modules that mirror research priorities of the VA and HSR&D and make it possible to leverage these DIPEx modules to inform future research and to act as a resource to extend other forms of engagement.

## VA HER ACTIVITIES

The HER subgroup laid out steps for building capacity to produce broadly inclusive health experiences studies that capture the widest possible range of Veterans’ health experiences. Several of these steps have been achieved or are in progress, including the development of a website for disseminating DIPEx modules; the launch of the first VA-funded DIPEx module on Veterans with Traumatic Brain Injury;^20^ and securing funding for two additional high-priority-area VA modules: Veteran Experiences with Gulf War Illness (GWI), and Firearm Injuries Among Rural Veterans.

Themes identified in the TBI and GWI modules highlight the range of individual, relational, health systems, and societal domains impacting Veteran health experiences. Many Veterans who participated in these modules were confronted with a confluence of wartime exposures or injuries that led to physical and mental challenges, social and occupational disruption, and issues re-integrating into civilian life. Veterans identified barriers to seeking healthcare including being influenced by a military culture of not complaining or seeking care, having mistrust in the medical system, and challenges with navigating medical systems. Importantly, across the TBI and GWI modules, Veterans offered important insights related to future research, advice for VA clinicians, and messages for others.^18, 20^ Participants expressed the value of telling their story; some told us this was the first time someone had listened to their entire story, others were thankful for the opportunity to share their experiences in a way that might help others (Table 2). Contrary to other forms of qualitative work, data from hard-to-reach populations organized in DIPEx modules are broadly disseminated and available for future use. For example, in the TBI and GWI modules, female Veterans are represented in higher proportions than expected, given relatively lower numbers of female service members. This multimodal approach provides a rich, ongoing resource that includes unfiltered voices and experiences Veterans for multiple potential future uses.

**Table 2:** Quotes from Modules Exemplifying Participant Perspectives on Value of Modules

Module	Quote
Gulf War Veteran #1	*People’s stories need to be heard, our stories need to be heard, and the truth will set you free.*
Gulf War Veteran #2	*As a female in the military, ex-military and the VA world, women need to be advocates for women. Because there’s all these services but most all of them are all geared around men, not that they are gender labelled, but it’s just known that most men, that men are in the military.*
Caregiver of Gulf War Veteran	*Gulf War illness is extremely difficult on the family all of those things are what our story is. And you’re not alone, that’s happening, it’s Gulf War illness and that’s why I want to tell our story because I would have loved to have heard my story and been able to relate.*
Veteran with TBI #1	*For one thing, I don’t want these kids now ever go through the same, same stuff I did. And a lot of us Vietnam Vets are – I can’t speak for the whole, whole demographic group, but there’s a lot of us that feel the same way, and we’re helping these guys coming back because we don’t want them to go through the ignorance and or abuse that we did. And, and I help them in that, you know, like I said about the, about the benefits, the, the VA has. Not, not just for PTSD – housing, education, the whole gamut.*
Veteran with TBI #2	*A counselor said once to me, because I kept saying “I just, I just want to be normal. I just want to be normal. I’m tired of being like this.” And she was like “Well, what you were experiencing was not abnormal. You were having a perfectly normal reaction to a highly abnormal situation.” And that was like, just a lightbulb for me of like, what I’m going through is not abnormal. I’m not, I’m not broken. I’m not odd. I’m not alone in this. Like there are other people having the same reaction. This is a normal reaction to have to seeing what we saw. Trying to explain that to my family is challenging. Trying to explain to anybody who hasn’t seen it or isn’t well versed in the treatment of it is, is difficult. Like, even people out in the community are kind of like, “I don’t understand why you guys are like that.” Well I mean, “Cause you weren’t there.”*

## ROADMAP FOR FUTURE DIRECTIONS

The HER subgroup recommended several steps to continue developing the use of HER in VA. First, the development of a more systematic way to select priorities for module topics that are consistent with VA and HSR&D priorities and inform a wide range of services and conditions.^1^ Second, the creation of a network of researchers and partners.^1^ For the VA module topics thus far, collaborations have been formed across two VA Health Care Systems and three academic affiliates, and several investigators at each of these institutions have been trained in DIPEx methodology. With the creation of each module, a network of clinician, scientist, and Veteran advisory panels are formed, and different Veteran advocacy groups are mobilized to help with recruitment and dissemination of the final module product. The final recommendation from the HER subgroup was to “pilot the use of modules in planning other research.” The importance is being explored. By contributing to a future-use repository, the rich data collected using the DIPEx methodology can be used for additional purposes, including designing future studies, rather than be destroyed.^19^ A critical next step is evaluating the use of DIPEx web-based modules and the VA repository for informing the conception, design, and implementation of VA studies.

## CONCLUSION

The DIPEx methodology for conducting HER is a rigorous qualitative approach, geared toward understanding people’s experiences of health and illness, including experiences from under-represented populations who may be less likely to participate in other forms of research or engagement. Many components of DIPEx provide a valuable resource for learning about a broad, often difficult to access, range of experiences. At its core, DIPEx places a high value on voices being heard, and engenders a commitment to listening, which is the ultimate form of engagement and is the foundation of trust. Valuing people’s stories and perspectives—and making them accessible to others—allows stories to become influential, giving ideas to lay persons, clinicians, and researchers, ultimately help find solutions.
